# Pathway and kinetics of cyhalothrin biodegradation by *Bacillus thuringiensis* strain ZS-19

**DOI:** 10.1038/srep08784

**Published:** 2015-03-05

**Authors:** Shaohua Chen, Yinyue Deng, Changqing Chang, Jasmine Lee, Yingying Cheng, Zining Cui, Jianuan Zhou, Fei He, Meiying Hu, Lian-Hui Zhang

**Affiliations:** 1 Guangdong Province Key Laboratory of Microbial Signals and Disease Control, South China Agricultural University, Guangzhou 510642, Peoples' Republic of China; 2 Institute of Molecular and Cell Biology, Agency for Science, Technology and Research (A*STAR), 61 Biopolis Drive, Proteos, Singapore 138673, Republic of Singapore; 3 Key Laboratory of Natural Pesticide and Chemical Biology, Ministry of Education, South China Agricultural University, Guangzhou 510642, Peoples' Republic of China; 4 Department of Biological Sciences, National University of Singapore, Republic of Singapore

## Abstract

Cyhalothrin is a common environmental pollutant which poses increased risks to non-target organisms including human beings. This study reported for the first time a newly isolated strain, *Bacillus thuringiensis* ZS-19 completely degraded cyhalothrin in minimal medium within 72 h. The bacterium transformed cyhalothrin by cleavage of both the ester linkage and diaryl bond to yield six intermediate products. Moreover, a novel degradation pathway of cyhalothrin in strain ZS-19 was proposed on the basis of the identified metabolites. In addition to degradation of cyhalothrin, this strain was found to be capable of degrading 3-phenoxybenzoic acid, a common metabolite of pyrethroids. Furthermore, strain ZS-19 participated in efficient degradation of a wide range of pyrethroids including cyhalothrin, fenpropathrinn, deltamethrin, beta-cypermethrin, cyfluthrin and bifenthrin. Taken together, our results provide insights into the mechanism of cyhalothrin degradation and also highlight the promising potentials of *B.thuringiensis* ZS-19 in bioremediation of pyrethroid-contaminated environment. This is the first report of (i) degradation of cyhalothrin and other pyrethroids by *B.thuringiensis*, (ii) identification of 3-phenoxyphenyl acetonitrile and *N*-(2-isoproxy-phenyl)-4-phenoxy-benzamide as the metabolites in the degradation pathway of pyrethroids, and (iii) a pathway of degradation of cyhalothrin by cleavage of both the ester linkage and diaryl bond in a microorganism.

Cyhalothrin [(*RS*)-*α*-Cyano-3-phenoxybenzyl-(*Z*)-(1*RS*,3*RS*)-(2-chloro-3,3,3-trifluoro propenyl)-2,2-dimethylcyclopropanecarboxylate] is one of the main pyrethroids, which are widely used in agriculture, forestry, horticulture, public health (e.g. hospitals and construction sites) and homes for the control of a broad spectrum of insect pests^1^. Over the past several decades, the usage of cyhalothrin has been gradually increasing globally, especially with the phaseout of organophosphates use in residential home and some agricultural applications^2^. Unfortunately, extensive use of cyhalothrin has resulted in serious environmental contamination problems^3^. Numerous reports revealed cyhalothrin is ubiquitous in water sources from either residential or agricultural runoff^4^,^5^,^6^,^7^. As a result, humans have an increased risk of exposure to cyhalothrin. The pesticide enters humans via ingestion of food or drinking water or inhalation, or dermal contact^8^,^9^,^10^.

Although cyhalothrin has relatively low mammalian toxicity, there is still caution with regard to human exposure^8^,^11^. A number of studies have demonstrated that large dose exposures in mammals may cause significant toxicity and health effects, including neurotoxicity^12^, genotoxicity^13^,^14^, cytotoxicity^14^,^15^, and endocrine disruption which can damage mammalian reproduction^16^,^17^,^18^. Furthermore, chronic exposure to cyhalothrin even low level exposures may be associated with an elevated risk of mutagenicity^19^, carcinogenicity^20^, as well as childhood leukemia^21^. Additionally, cyhalothrin is also highly toxic to aquatic invertebrates and fish^22^. Its half-life varies from 17 to 110 days in water^23^. The large amounts of evidence suggest cyhalothrin has posed a great threat to human health and ecosystems^24^. Therefore, the need for effective strategies to remove cyhalothrin from environment is urgent.

Several conventional methods such as photodecomposition, fenton degradation, ozonation, adsorption and incineration have been used for treatment of organic pollutants^25^,^26^. However, these physicochemical methods are expensive and not environment friendly due to the release of hazardous materials as by-products^27^,^28^. Recently, biodegradation has emerged as a great potential alternative approach to control pesticide residues because of its cost-effective and eco-friendly properties^29^,^30^. Currently, a few pyrethroid biodegradation mechanisms have been studied, such as pyrethroid-degrading strains, *Serratia* sp. JCN13^31^, *Streptomyces parvulus* HU-S-01^32^, and *Brevibacterium aureum* DG-12^33^, and the three genes, i.e., *Estp*, *pytH*, and *PytZ*, encoding pyrethroid-hydrolyzing carboxylesterases from *Klebsiella* sp. ZD112^34^, *Sphingobium* sp. JZ-1^35^, and *Ochrobactrum* *anthropi* YZ-1^36^, respectively. In addition, one new monooxygenase CMO involved in pyrethroid degradation has recently been purified and identified from a *Streptomyces* sp.^37^. However, there is no report of complete degradation of cyhalothrin by bacterial isolates. Moreover, the biodegradation pathway of cyhalothrin has not been investigated so far and remains unknown.

The objectives of the present study were: (1) to isolate a promising bacterial strain for the treatment of cyhalothrin-contaminated environment; (2) to determine the kinetic parameters for the biodegradation of cyhalothrin and other pyrethroids; and (3) to elucidate the biodegradation mechanism of cyhalothrin by the isolate.

## Results

### Isolation and identification of cyhalothrin-degrading bacterium

A highly efficient cyhalothrin-degrading bacterial strain, designated as ZS-19, was isolated from an activated sludge sample collected from a pyrethroid-contaminated area using the enrichment method. The isolate utilized cyhalothrin as the carbon and nitrogen source in minimal medium (MM), and completely degraded the pesticide within 72 h.

**Figure 1:**
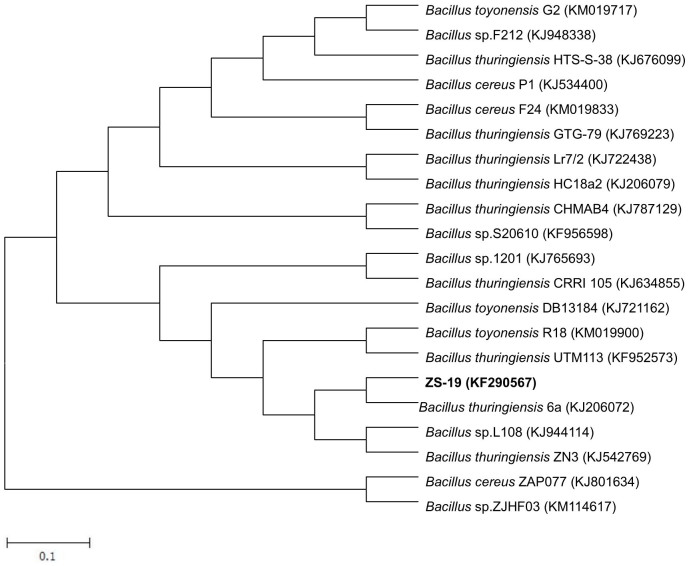
Phylogenetic tree based on 16S rDNA sequences of strain ZS-19 and the related *Bacillus* species. The numbers in parentheses represent the sequence accession number in GenBank. Bar represents sequence divergence.

Strain ZS-19 produced clear haloes around the colonies when grown on agar plate containing 100 μg·ml^−1^ cyhalothrin (Figure S1). It is a gram-positive, obligately aerobic, rod-shaped bacterium with dimensions of 2.5 to 3.5 μm in length and 0.5 to 1.0 μm in width. Colonies grown on Luria-Bertani (LB) agar plates were big, white or slightly yellow, opaque, and rough with irregular margin. This bacterium showed a positive reaction in glucose, ribose, trehalose, etc, but negative in erythritol, adonitol, galactose, etc. The detailed physio-biochemical characteristics of strain ZS-19 are presented in Table 1. Phylogenetic analysis of the 16S rDNA gene sequences indicated that strain ZS-19 was grouped among *Bacillus* species and closely clustered with *Bacillus thuringiensis* strain 6a (GenBank accession number KJ206072) with identity of 99% (Figure 1). The bacterium was further classified as *B.thuringiensis* by API 50 CH system, with high identification (99.2%). Based on the morphology, physio-biochemical properties, and 16S rDNA gene analysis as well as API identification systems, strain ZS-19 was confirmed as *B.thuringiensis.* The partial 16S rDNA gene sequence of *B.thuringiensis* strain ZS-19 was deposited in GenBank under the nucleotide accession number KF290567. Degradation of cyhalothrin by *B.thuringiensis* is the first ever report.

### Growth and degradation studies using cyhalothrin

**Figure 2:**
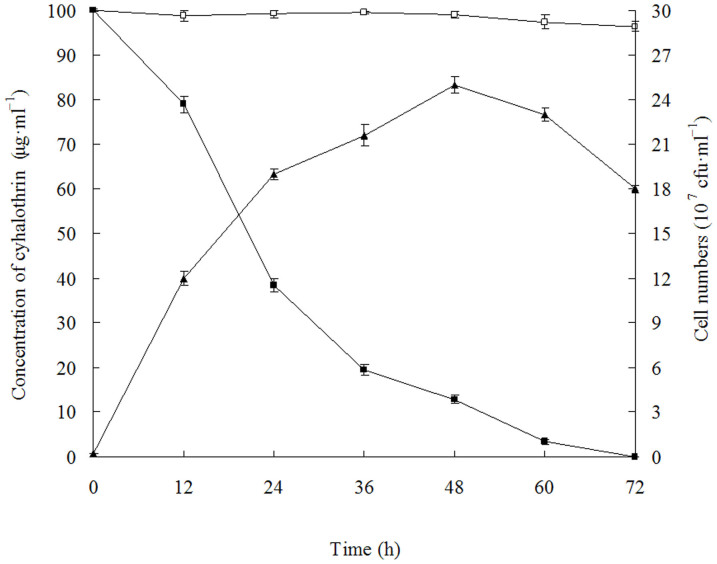
Degradation and utilization of cyhalothrin (100 μg·ml^−1^) during growth of strain ZS-19. Symbol: □, cyhalothrin control; ▪, cyhalothrin degradation by strain ZS-19; ▴, cell growth. Data represent mean values of three replicates with standard deviation.

The growth of strain ZS-19 with cyhalothrin as the growth substrate in MM medium and the kinetics of cyhalothrin degradation are shown in Figure 2. In the initial cultivation phase (0–24 h), the number of bacterial cells and cyhalothrin biodegradation all exhibited rapid increase trends, and the cyhalothrin biodegradation reached 61.5% at 24 h. At 48 h post incubation, the number of bacterial cells was increased to its maximum level, then the number was gradually decreased. Strain ZS-19 degraded cyhalothrin completely within 72 h. In contrast, when inoculated into LB medium, strain ZS-19 degraded only 86.0% cyhalothrin (100 μg·ml^−1^) at 72 h post inoculation, and complete degradation occurred at 120 h (data not shown). High performance liquid chromatography (HPLC) analysis of cyhalothrin degradation by strain ZS-19 over time is shown in Figure S2. In the control experiment, no significant change in cyhalothrin concentration was observed in the non-inoculated medium.

### Substrate inhibition studies

**Figure 3:**
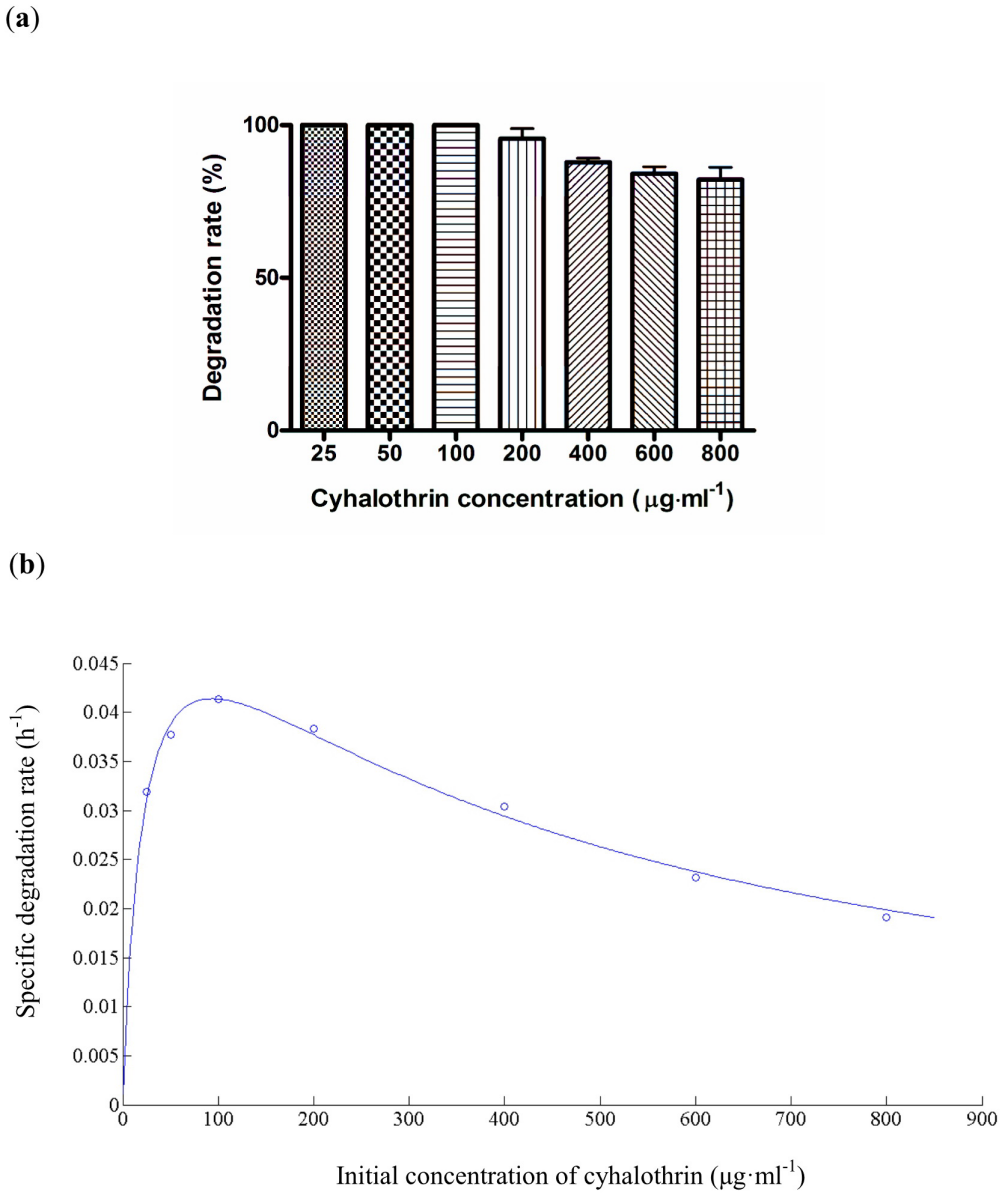
(a) Cyhalothrin degradation by using different initial cyhalothrin concentrations by strain ZS-19 within 72 h. Data represent mean values of three replicates with standard deviation. (b) Relationship between initial cyhalothrin concentration and specific degradation rate by strain ZS-19.

The cyhalothrin biodegradation by using different initial cyhalothrin concentrations is shown in Figure 3a. Strain ZS-19 utilized and degraded cyhalothrin in a concentration as high as 800 μg·ml^−1^, and no lag period was observed. When the initial cyhalothrin concentration was <100 μg·ml^−1^, cyhalothrin was completely degraded within 72 h. When the initial cyhalothrin concentration was increased to 200, 400, 600, and 800 μg·ml^−1^, respectively, approximately 95.5%, 87.4%, 84.0%, and 82.1% biodegradation of the pesticide was observed in 72 h, respectively. The results demonstrated that strain ZS-19 possessed good degradation performance on cyhalothrin.

It was observed that the biodegradation rate decreased following an increase in the initial cyhalothrin concentration, indicating that cyhalothrin may act as a partial inhibitor to strain ZS-19. To address this possibility, the substrate inhibition model was explored to fit the specific degradation rate (*q*) at different initial concentrations. Figure 3b showed the relationship between initial cyhalothrin concentration and specific degradation rate. The kinetic parameters including maximum specific degradation rate (*q*_max_), half-saturation constant (*K*_s_) and inhibition constant (*K*_i_) for the substrate inhibition model were determined to be 0.0614 h^−1^, 22.6672 μg·ml^−1^ and 387.6156 μg·ml^−1^, respectively using non-linear regression analysis by Matrix Laboratory (MATLAB) software package. The critical inhibitor concentration (*S*_m_) was calculated to be 93.7345 μg·ml^−1^. The correlation coefficient (*R*^2^) was 0.9902, which indicates that the calculated values of the model were in perfect agreement with the experimental values. As shown in Figure 3b, when the initial content of cyhalothrin was >93.7345 μg·ml^−1^, *q* value was gradually decreased, suggesting that the cyhalothrin biodegradation activity could be partially inhibited at a high concentration of cyhalothrin but may not lead to a complete repression.

**Figure 4:**
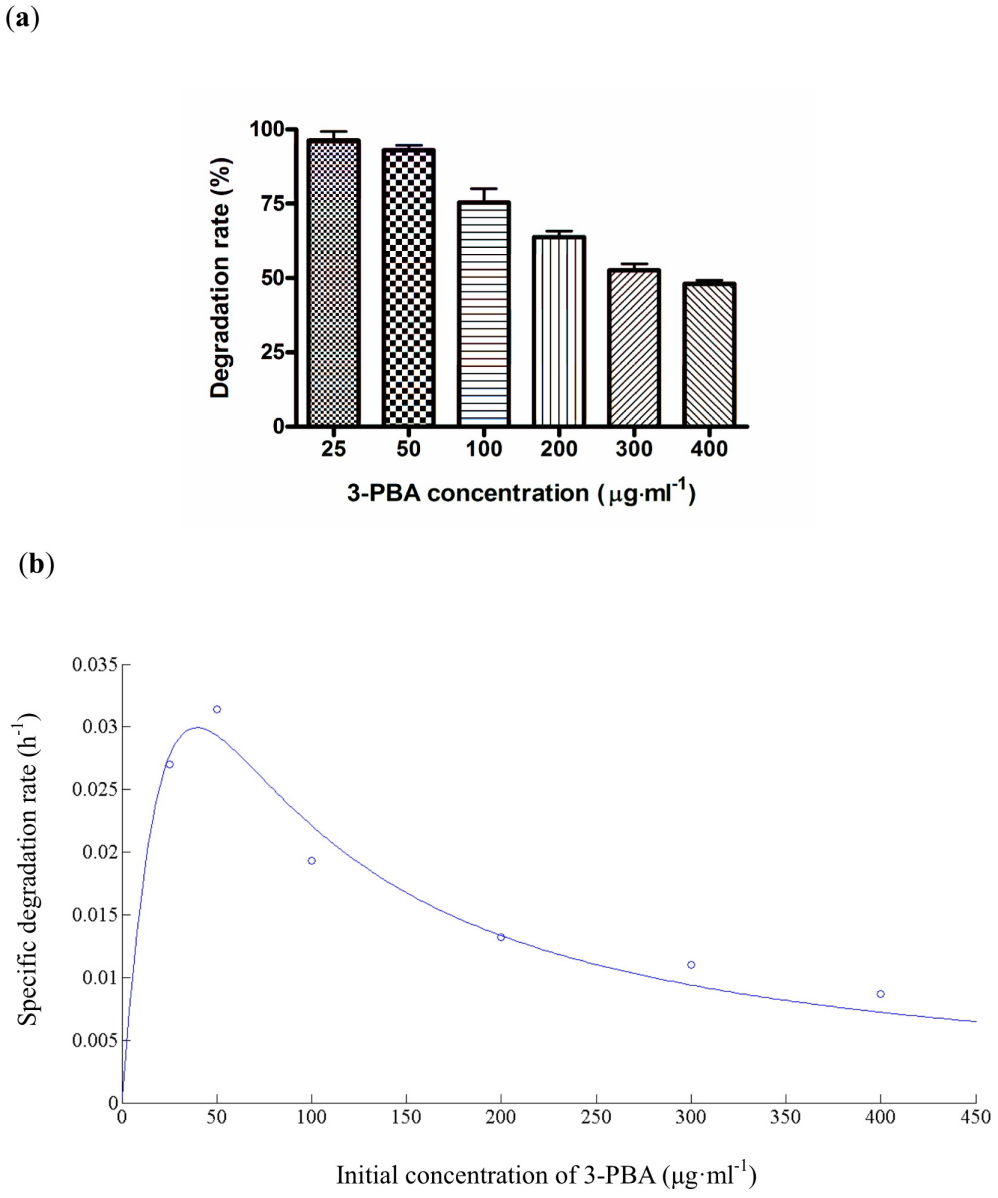
(a) 3-PBA degradation by using different initial 3-PBA concentrations by strain ZS-19 within 72 h. Data represent mean values of three replicates with standard deviation. (b) Relationship between initial 3-PBA concentration and specific degradation rate by strain ZS-19.

3-Phenoxybenzoic acid (3-PBA) is a common product of pyrethroids in the environment^38^. To test the effect of 3-PBA concentration on its degradation, strain ZS-19 was inoculated to the media containing various initial 3-PBA concentrations varying from 25 to 400 μg·ml^−1^. Strain ZS-19 rapidly degraded and utilized the added 3-PBA up to a concentration of 400 mg·L^−1^. When the initial 3-PBA concentration was <50 μg·ml^−1^, degradation got over 90% in 72 h. However, along with the increased concentration of the compound, the rate of degradation was slowdown, and the percentage of 3-PBA removal reached about 75.4%, 63.8%, and 52.5% at the concentration of 100, 200, and 300 μg·ml^−1^, respectively. When the initial 3-PBA concentration was increased to 400 μg·ml^−1^, only 48% 3-PBA was degraded (Figure 4a). These findings reveal that increased concentration of 3-PBA has a marked effect on biodegradation performance of strain ZS-19, but did not lead to complete inhibition similar to cyhalothrin biodegradation.

The relationship between initial 3-PBA concentration and specific degradation rate is given in Figure 4b. The kinetic parameters *q*_max_, *K*_s_ and *K*_i_ were established to be 0.1270 h^−1^, 64.0577 μg·ml^−1^ and 24.3686 μg·ml^−1^, respectively. The value of *R*^2^ was 0.9623 indicating that the experimental data were well correlated with the inhibition model. The critical inhibitor concentration was determined to be 39.5094 μg·ml^−1^, suggesting that when the initial concentrations of 3-PBA were <39.5094 μg·ml^−1^, the *q* value was gradually increased. At higher concentrations, inhibition by 3-PBA became substantial and *q* value was proportionally decreased in a dosage dependent manner.

### Identification of metabolites

The HPLC and gas chromatography-mass spectrometry (GC-MS) studies were conducted to monitor the degradation of cyhalothrin by strain ZS-19. HPLC analysis confirmed complete degradation of cyhalothrin by strain ZS-19 within 72 h (Figure S2). To identify the metabolite formed during the degradation of cyhalothrin by strain ZS-19, GC-MS was carried out. In the sample of 12 h, a significant peak was detected, showing a characteristic mass fragment [M^+^] at *m*/*z* 450 with major fragment ions at *m*/*z* 141 and 181, which are similar to characteristic parental ions of cyhalothrin (Figure S3a). The retention time (RT) of the compound was 27.870 min, which exactly matched with the authentic standard of cyhalothrin in the National Institute of Standards and Technology (NIST, USA) library database. Thus, the compound was identified as cyhalothrin (Figure S4a). Along with the degradation process, the parent compound disappeared concomitantly with formation of six new metabolites, which were characterized as *α*-hydroxy-3-phenoxy-benzeneacetonitrile, 3-phenoxyphenyl acetonitrile, *N*-(2-isoproxy-phenyl)-4-phenoxy-benzamide, 3-phenoxybenzaldehyde, 3-phenoxybenzoate, and phenol, respectively (Figure S4b–g), based on the similarity of their fragment retention times and molecular ions to those of corresponding authentic compounds in the NIST library database (Figure S3b–g). The chemical name in NIST library, retention times and characteristic ions of *m*/*z* are presented in Table 2. Among these identified metabolites, 3-phenoxyphenyl acetonitrile and *N*-(2-isoproxy-phenyl)-4-phenoxy-benzamide were observed for the first time in the degradation pathway of pyrethroids. We also noticed that all these metabolites were transient and they disappeared gradually. No persistent accumulative metabolite was detected after dissipation of the parent compound.

**Figure 5:**
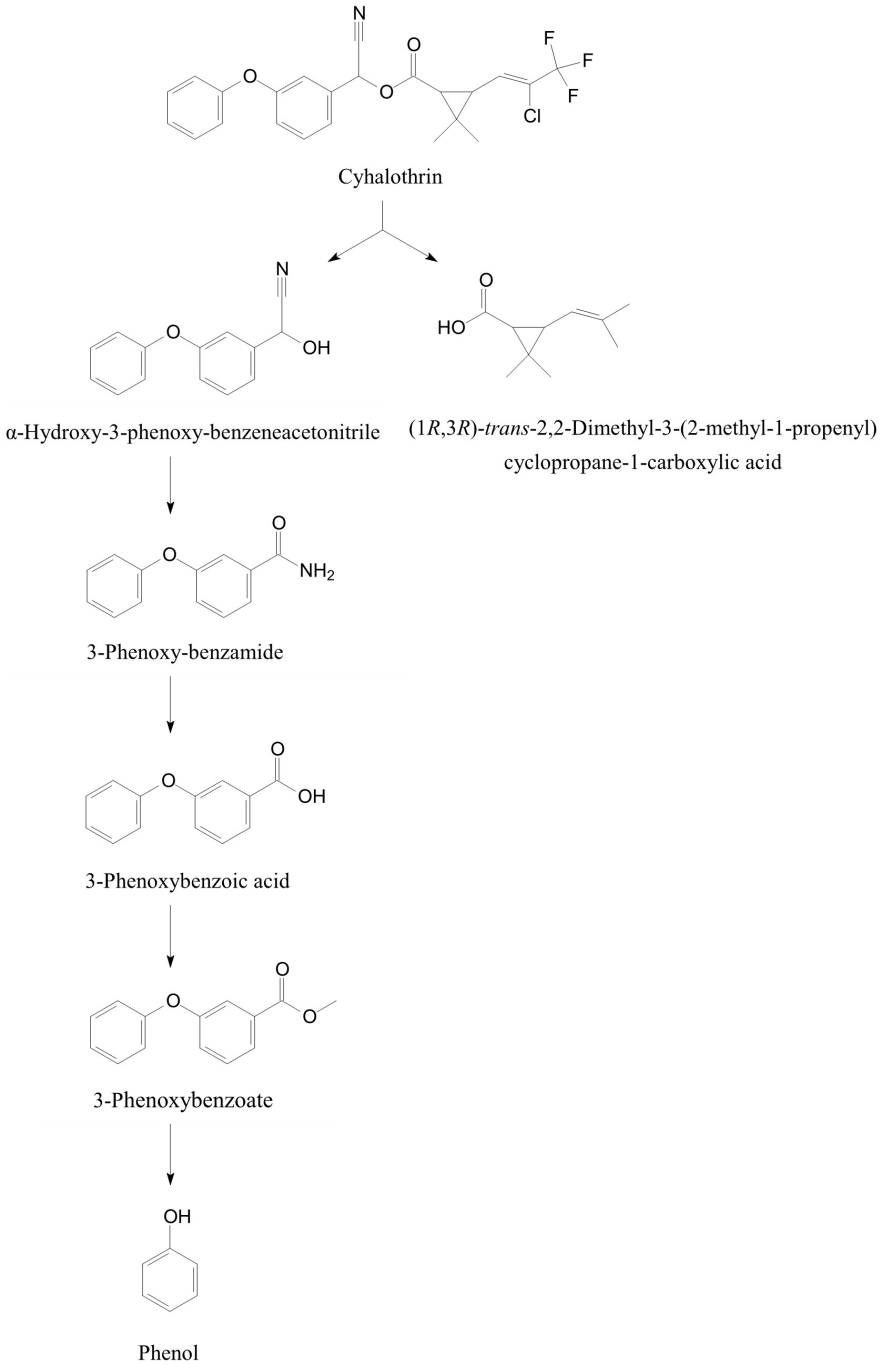
Proposed pathway for degradation of cyhalothrin in strain ZS-19.

On the basis of chemical structures of cyhalothrin and the identified metabolites, a novel degradation pathway of cyhalothrin was proposed in strain ZS-19 (Figure 5). Cyhalothrin was initially degraded via hydrolysis, resulting in cleavage of the ester linkage. Then, cyhalothrin was further transformed by cleavage of diaryl bond, followed by degradation of the aromatic ring and subsequent metabolism. Eventually, cyhalothrin was degraded by strain ZS-19 without any persistent accumulative product. Therefore, we deduce the bacterial strain may harbor a complete metabolic pathway for degradation and metabolism of cyhalothrin. To our knowledge, this is the first report of cyhalothrin degradation pathway in a microorganism.

### Biodegradation kinetics of various pyrethroids

**Figure 6:**
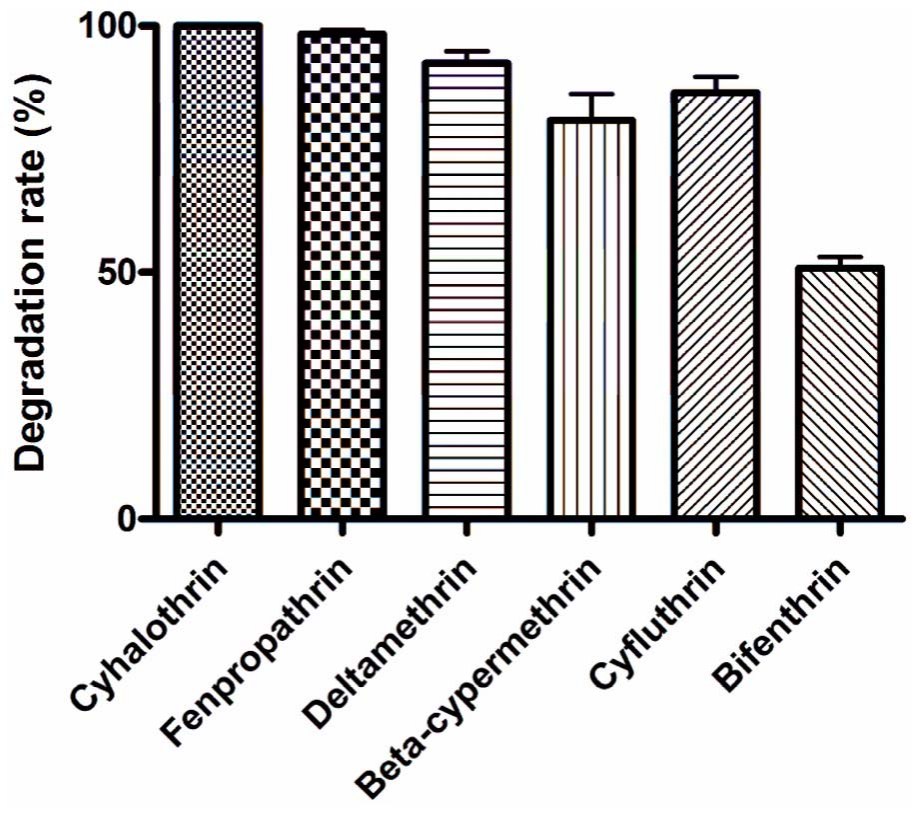
Degradation rate of various pyrethroids by strain ZS-19 within 72 h. Data represent mean values of three replicates with standard deviation.

The abilities of strain ZS-19 to degrade various pyrethroids were tested in this study. Cyhalothrin, fenpropathrin, deltamethrin, beta-cypermethrin, cyfluthrin, and bifenthrin were all efficiently degraded by strain ZS-19, with a degradation rate of 100%, 98.3%, 92.4%, 80.8%, 86.4%, and 50.9% within 72 h, respectively (Figure 6). Strain ZS-19 displayed no obvious substrate specificity for different pyrethroids. As shown in Figure S5, biodegradation of all the tested compounds started rapidly at the beginning of incubation with no apparent lag phase.

To further quantify the degradation efficiency of various pyrethroids by strain ZS-19, the degradation rate constant (*k*) and half-life (*t*_1/2_) from the first-order kinetic model were calculated. Kinetic data showed that the degradation process followed the first-order kinetic model, with a correlation coefficient (*R*^2^) varying from 0.9196 to 0.9641, suggesting that the experimental data were well-correlated with the model. The biodegradation process was characterized by a *k* ranging from 0.0112 to 0.0413 h^−1^. The *t*_1/2_ value was determined to be 16.8 to 61.9 h, which was sharply shortened as compared to those in the environment. The kinetic parameters for degradation of various pyrethroids by strain ZS-19 are tabulated in Table 3.

## Discussion

*Bacillus thuringiensis* strain ZS-19 was isolated from a pyrethroid-contaminated site by using enrichment culture technique, and it was found to be highly effective in degrading cyhalothrin and other pyrethroids in this study. *B.thuringiensis* (Bt) is a well-known bacterium for its broad capabilities, which has become the main microorganism widely used in biological control^39^. With the growing demand for food free of chemical pesticides, the application of Bt to combat insect pests and plant diseases of human interest has gained momentum^40^,^41^. However, the potential use of Bt in bioremediation of environmental pollutants has not received the attention it deserves. Even though it has been reported that some Bt strains have the potential of degrading xenobiotic compounds such as chlorpyrifos^42^, dimethyl phthalate^43^, and acid red 119^44^, thus far, there has been no report of biodegradation of cyhalothrin and other pyrethroids by Bt isolates. This study provides the first evidence that Bt strain ZS-19 participates in efficient degradation of a wide range of pyrethroids, which are all extensively used insecticides with environmental contamination problems^2^,^3^,^4^,^5^,^6^,^7^. This discovery demonstrates the potential of the microorganism and new opportunities opening for future applications.

It is worth mentioning that strain ZS-19 utilized cyhalothrin as the carbon and nitrogen source, and completely degraded the pesticide within 72 h. Although several pyrethroid-degrading bacterial strains have been isolated^33^,^45^,^46^,^47^,^48^; however, there is no report of complete degradation of cyhalothrin by any bacterial isolates. Another important feature of this particular bacterium is that it rapidly degraded cyhalothrin up to a concentration of 800 μg·ml^−1^ with no lag phase. It's observed that high concentration of cyhalothrin showed a limited effect on the biodegradation and it did not lead to a complete repression (Figure 3). This is a contrast to the previous studies of Jilani and Khan who reported that increased concentration of pyrethroid had a marked effect on the biodegradation performance of *Pseudomonas* sp. IES-Ps-1 with a modest increased in the duration of lag phase^49^. Our results suggest that *B.thuringiensis* strain ZS-19 may be suitable for bioremediation of various contaminated environments.

In addition to degradation of cyhalothrin, *B.thuringiensis* strain ZS-19 was found to be highly effective in degrading a wide range of pyrethroids including fenpropathrinn, deltamethrin, beta-cypermethrin, cyfluthrin, and bifenthrin with the degradation process following the first-order kinetic model (Figure 6) (Table 3). The degradation half-lives of various pyrethroids by strain ZS-19 were calculated to be 16.8–61.9 h, which are drastically shortened as compared to the reported *t*_1/2_ for pyrethroids in the environment varying from 17 to 600 days^23^,^50^. This is an important feature of a microorganism to be employed for bioremediation of pyrethroid-contaminated environments because various regions are usually affected by multiple pyrethroid compounds^2^,^4^,^5^,^6^,^7^. More importantly, strain ZS-19 was capable of degrading 3-phenoxybenzoic acid (3-PBA), a common metabolite of pyrethroids^38^. Simultaneous degradation of pyrethroids and their metabolite 3-PBA in a microorganism is rarely seen in other bacterial isolates. Liu and co-workers reported that *B.licheniformis* B-1 degraded only 50.36% of cypermethrin at 72 h and enhanced degradation could not occur due to the formation of 3-PBA in the medium^51^. Interestingly, three strains of *Pseudomonas* spp. could utilize 3-PBA as a growth substrate in soils; however, it is not clear whether these microorganisms are able to degrade pyrethroid compounds^52^. 3-PBA is considered far more potent as an endocrine disruptor than the parent molecules^53^. Owing to its potential antimicrobial activities, 3-PBA is not only refractory to microbial attack but also limits the further biodegradation of pyrethroids^53^,^54^. Therefore, degradation of this compound by the same microorganism that degrades pyrethroids is critically important.

**Figure 7:**
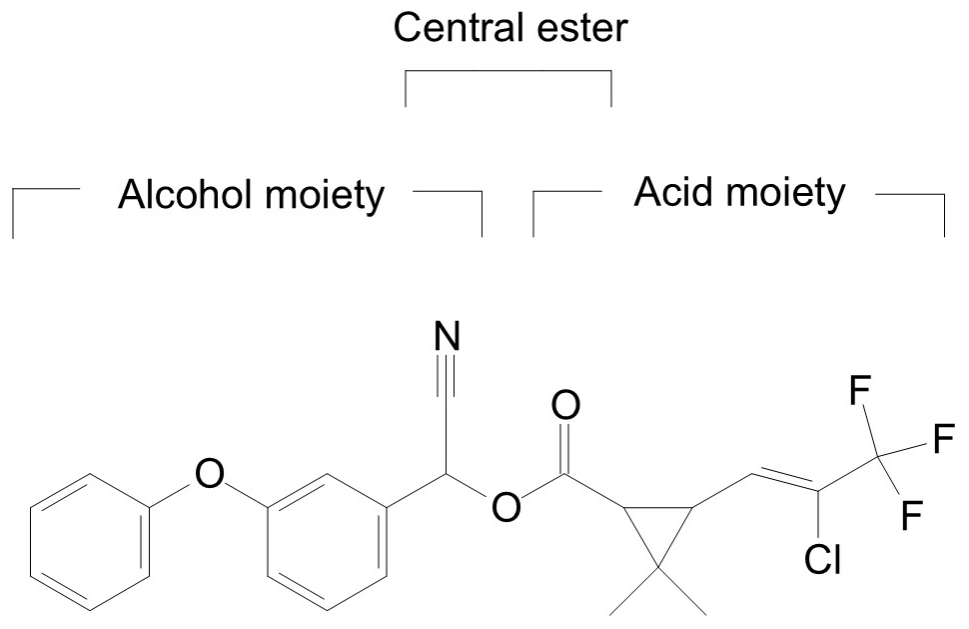
Chemical structure of cyhalothrin.

It is generally acknowledged that ester hydrolysis via carboxylesterases is the primary step of degradation and detoxification of various pyrethroids in a multitude of species, from mammals, insects to bacteria^31^,^35^,^36^,^55^,^56^. Several pyrethroid biodegradation pathways have been proposed in a few microorganisms^33^,^57^,^58^,^59^,^60^. *Micrococcus* sp. CPN 1 degraded cypermethrin by hydrolysis of ester linkage to yield 3-phenoxybenzoate that was further metabolized to form protocatechuate and phenol via diphenyl ether cleavage^57^. *Streptomyces parvulus* HU-S-01 converted cypermethrin to 3-PBA and dichloroving acid through hydrolysis, but it could not further transform the degradation products^32^. Unfortunately, there is no report of cyhalothrin degradation pathway in a microorganism. In this study, the metabolites of cyhalothrin by *B.thuringiensis* strain ZS-19 were confirmed in order to elucidate the cyhalothrin biodegradation mechamism. Six metabolites were detected as the intermediate products during cyhalothrin degradation (Table 2) (Figure S3) (Figure S4). Among them, *α*-hydroxy-3-phenoxy-benzeneacetonitrile, 3-phenoxybenzaldehyde, 3-phenoxybenzoate and phenol have been reported in the degradation pathway of other pyrethroids^33^,^57^,^58^,^60^. Interestingly, 3-phenoxyphenyl acetonitrile and *N*-(2-isoproxy-phenyl)-4-phenoxy-benzamide were first identified in the degradation pathway of pyrethroids, indicating strain ZS-19 may have a different cyhalothrin degradation pathway from the reported pyrethroid pathways^33^,^57^,^58^,^59^,^60^. On the basis of chemical structures of cyhalothrin and the identified metabolites, we concluded that the degradation of cyhalothrin by strain ZS-19 was initiated by cleavage of the carboxylester linkage through hydrolysis, similar to that presented in mammals and insects^55^,^56^. Hydrolysis is the main mechamism during the cyhalothrin degradation by strain ZS-19. This could be due to the fact that cyhalothrin is an ester compound (Figure 7), which is susceptible to attack via hydrolysis. Significantly, in addition to hydrolysis of carboxylic ester, strain ZS-19 further metabolized these intermediates by cleavage of the diaryl bond and aromatic ring. Thus, a novel degradation pathway of cyhalothrin in strain ZS-19 was proposed based on analysis of the metabolites (Figure 5). This is the first report of a pathway of degradation of cyhalothrin by hydrolysis of ester linkage and cleavage of diaryl bond and aromatic ring in a microorganism, which we propose is of vital importance in cyhalothrin biogeocycle.

## Methods

### Isolation of cyhalothrin degraders

Samples from the activated sludge in a pyrethroid-manufacturing wastewater treatment system were suspended in minimal medium (MM) [(per litre contains 10.5 g K_2_HPO_4_, 4.5 g KH_2_PO_4_, 2 g (NH_4_)_2_SO_4_, 2 g glycerol, 0.2 g MgSO_4_·7H_2_O, 5 mg FeSO_4_, 10 mg CaCl_2_, and 2 mg MnCl_2_, pH 7.5] containing cyhalothrin (100 μg·ml^−1^). The culture was incubated under aerobic conditions at 30°C. At 7 days post incubation, portions (10%, *v*/*v*) were transferred into another fresh MM containing cyhalothrin (200 μg·ml^−1^) and incubated for another 7 days. After several serial transfers, samples were spread on MM agar (1.8%) plates containing cyhalothrin (100 μg·ml^−1^). Individual colonies were transferred into 50 ml of MM containing cyhalothrin (100 μg·ml^−1^) as the carbon and nitrogen source. Cyhalothrin concentrations in the culture fluids were extracted and determined by high performance liquid chromatography (HPLC) (Waters, USA) at appropriate intervals. One pure isolate designated ZS-19 showing the highest degradation activity was selected for further study.

### Bacterial identification

The degrader was identified by morphology, physio-biochemical characteristics, and genetic analysis based on 16S rDNA gene sequence as well as API identification systems. Colony morphology was observed on Luria-Bertani (LB) (per litre contains 10.0 g tryptone, 5.0 g yeast extract, and 10.0 g NaCl, pH 7.5) agar plates incubated at 30°C. Cell morphology was observed with scanning electron microscopy (XL-30 ESEM, Philips Optoelectronics Co., Ltd, Holland). Genomic DNA was prepared with a MasterPure™ DNA Purification Kit (Epicentre Biotechnologies, USA) according to the protocols of the manufacturer. The 16S rDNA gene was PCR amplified with universal primers as described previously^33^. PCR products were purified with a QIAquick PCR Purification Kit (QIAGEN) and sequenced by Institute of Molecular and Cell Biology (Proteos, Singapore). The resulting 16S rDNA gene sequences (1245 bp) were compared with the sequences in the GenBank nucleotide library using BLAST program. Multiple sequence alignment was carried out using Clustal X 1.8.1 and phylogeny was analyzed using MEGA 4.0. An unrooted tree was constructed using the neighbor-joining method. Finally, the isolate was further confirmed by API 50 CH system (bioMérieux Inc., France) according to the manufacturer's directions.

### Inoculum preparation

The bacterial strain was stored in 15% glycerol at −80°C. Before each experiment the strain was thawed and inoculated into a 250-ml Erlenmeyer flask, which contained 50 ml of sterile LB medium. Then the flask was placed on a platform shaker at 180 *g* and 30°C. The bacterial cells in the late-exponential growth phase were harvested by centrifugation (5 min, 4000 × *g*) at 4°C and washed twice in sterile *N*-saline (0.9% NaCl). Then, the washed strain was collected and suspended in sterile *N*-saline to achieve a cell density of about 1.5 × 10^8^ cells ml^−1^. The bacterial suspension was used as inoculum for the subsequent studies.

### Biodegradation assays

For the growth and degradation experiments, triplicate cultures were grown in MM containing **100** μg·ml^−1^ of cyhalothrin as the carbon and nitrogen source at 30°C and 180 *g* on a rotary shaker. Non-inoculated samples were kept as control. The sampling was performed at regular intervals. The bacterial growth was monitored by counting the colony forming units (cfu·ml^−1^) of serial dilutions, and the amount of residual cyhalothrin was measured by HPLC as described below.

To test the effect of initial cyhalothrin concentration on its degradation, the bacterial strain was inoculated to the MM media containing various cyhalothrin concentrations ranging from 25 to 800 μg·ml^−1^. The cultures were incubated at 30°C and 180 *g* on a rotary shaker. Each treatment was conducted in triplicate and cyhalothrin residues were determined periodically. Non-inoculated samples were served as control. The effect of initial 3-phenoxybenzoic acid (3-PBA) concentration on biodegradation were also tested with different 3-PBA concentrations varying from 25 to 400 μg·ml^−1^.

The abilities of strain ZS-19 to degrade various pyrethroids were studied. The MM media were supplemented with cyhalothrin, fenpropathrinn, deltamethrin, beta-cypermethrin, cyfluthrin, and bifenthrin at 100 μg·ml^−1^, respectively, and incubated at 30°C and 180 *g* on a rotary shaker for 72 h. The experiment was conducted in triplicate with non-inoculated samples as control. The sampling was carried out at a 12-h time interval and the pesticide residues were measured by HPLC as described previously^33^.

### Identification of metabolites

To identify cyhalothrin and its metabolites during the biodegradation, the bacterial strain was grown in MM media containing 100 μg·ml^−1^ cyhalothrin. The non-inoculated samples containing the same amount of cyhalothrin were used as control. Samples were collected at appropriate intervals and centrifuged. The supernatant was extracted with ethyl acetate from samples after acidification to pH 2 with 2 M HCl. The organic layer was dehydrated, dried and re-dissolved in methanol according to the method described in a previous report^57^.

After filtration with 0.45 μm membrane (Millipore, USA), the samples were subjected to gas chromatography-mass spectrometry (GC-MS) (Agilent, USA). GC-MS analysis was performed on a HP-5MS capillary column (30.0 m × 250 μm × 0.25 μm) with an Agilent 6890 N/5975 GC-MS system equipped with auto-sampler, an on-column, split/splitless capillary injection system, and with array detection from 30–500 nm (total scan). The column temperature was held initially at 90°C for 2 min, and increased to 150°C at the rate of 6°C·min^−1^ for 1 min, then increased to 180°C at the rate of 10°C·min^−1^ for 4 min, and finally increased to 260°C at the rate of 20°C·min^−1^ for 10 min. The ionization energy was 70 eV, and the temperatures corresponding to transfer line and the ion trap were 280°C and 230°C, respectively. The injection volume was 1.0 μL with splitless sampling at 250°C. The carrier gas (Helium) flow rate was 1.5 ml·min^−1 33,60^. The cyhalothrin and degradation intermediates identified by mass spectrometry analysis were matched with authentic standard compounds from the National Institute of Standards and Technology (NIST, USA) library database.

### HPLC analysis

Pyrethroid quantification was carried on a reverse-phase column (Phenomenex Lunar 5 μm C_18_ 250 × 4.6 mm) with a Waters 2690 HPLC system equipped with a ternary gradient pump, programmable variable-wavelength UV detector, column oven and electric sample valve, with array detection from 190-400 nm (total scan) based on retention time and peak area of the pure standard. A mixture of acetonitrile and water at a ratio of 80:20 was used as the mobile phase. Injection volume was 10 μL, and flow rate was 1.0 mL·min^−1^. The detection wavelengths of cyhalothrin, fenpropathrin, deltamethrin, beta-cypermethrin, cyfluthrin and bifenthrin were 276.8, 276.8, 250.9, 276.8, 276.8 and 354 nm, respectively.

3-PBA quantification was also analyzed by HPLC. A mixture of acetonitrile and water at a ratio of 70:30 was used as the mobile phase at a flow rate of 1.0 ml·min^−1^. pH value of water was adjusted to 2.4 using phosphoric acid before mixture as described previously with modification^52^.

### Kinetic analyses

The kinetic parameters of biodegradation of cyhalothrin or 3-PBA with different initial concentrations were determined by the substrate inhibition model (Eq.1) adapted from Luong^61^.
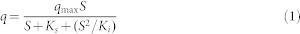
where *q* is the specific substrate degradation rate (h^−1^), *q*_max_ is the maximum specific substrate degradation rate (h^−1^), *K*_i_ is the substrate inhibition constant (μg·ml^−1^), *K*_s_ is the half-saturation constant (μg·ml^−1^), and *S* is the inhibitor concentration (μg·ml^−1^). The *q* value was determined from the gradient of a semi-logarithm plot of substrate concentration. From the value of *q* and the initial substrate concentration, the kinetic parameters including *K*_i_, *K*_s_ and *q*_max_ for substrate inhibition model were established using nonlinear regression analysis by Matrix Laboratory (MATLAB) software (Version 7.8). The critical inhibitor concentration (*S*_m_) was obtained from calculating the square root of *K*_i_ * *K*_s_.

Biodegradation process of various pyrethroids in liquid media was fitted to the first-order kinetic model (Eq.2) as described in detail previously^62^.
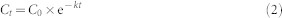
where *C*_0_ is the amount of substrate at time zero, *C*_t_ is the amount of substrate at time *t*, *k* and *t* are the degradation rate constant (h^−1^) and degradation period in hours, respectively.

The theoretical half-life (*t*_1/2_) values of different pyrethroids were calculated by the algorithm as expressed in Eq.3.
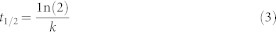
where ln (2) is the natural logarithm of 2, *k* is the degradation rate constant (h^−1^).

## Author Contributions

S.C., M.H. and L.H.Z. conceived and designed the experiments. S.C., Y.D., C.C., Y.C., Z.C., J.Z. and F.H. performed the experiments, analyzed the data, contributed reagents and materials. S.C., J.L. and L.H.Z. wrote the paper.

## Supplementary Material

Supplementary Information Supplementary Information

## Figures and Tables

**Table 1:** The physio-biochemical properties of strain ZS-19

Characteristics	Results (48 h)	Characteristics	Results (48 h)
Glycerol	+	Salicine	−
Erythritol	−	Cellobiose	+
D-Arabinose	−	Maltose	+
L-Arabinose	−	Lactose	−
Ribose	+	Melibiose	−
D-Xylose	−	Saccharose	−
L-Xylose	−	Trehalose	+
Adonitol	−	Inulin	−
Methyl *β*-D-xyloside	−	Melezitose	−
Galactose	−	Raffinose	−
Glucose	+	Amylose	+
Fructose	+	Glycogen	+
Mannose	−	Xylitol	−
Sorbose	−	Gentiobiose	+
Rhamnose	−	Turanose	−
Dulcitol	−	Lyxose	−
Inose	−	Tagatose	−
Mannitol	−	D-Fucose	−
Sorbitol	−	L-Fucose	−
Methyl *α*-D-mannoside	−	D-Arabitol	−
Methyl *α*-D-glucoside	−	L-Arabitol	−
*N*-acetyl glucosamine	+	Gluconate	+
Amygdalin	+	2-Keto-D-gluconate	−
Arbutin	+	5-Keto-D-gluconate	−
Esculin	+		

Note: +, tested positive; -, tested negative.

**Table 2:** Chromatographic properties of metabolites of cyhalothrin during degradation by strain ZS-19

Compound	RT (min)	*m*/*z*	Chemical name in NIST library
A	27.870	450	Cyhalothrin
B	17.590	225	*α*-Hydroxy-3-phenoxy-benzeneacetonitrile
C	21.550	209	3-Phenoxyphenyl acetonitrile
D	26.271	347	*N*-(2-isoproxy-phenyl)-4-phenoxy-benzamide
E	17.622	198	3-Phenoxybenzaldehyde
F	20.577	228	3-Phenoxybenzoate
G	6.051	94	Phenol

**Table 3:** Kinetic parameters for degradation of various pyrethroids by strain ZS-19

Pyrethroids	Regression equation	*k* (h^−1^)	*t*_1/2_ (h)	*R*^2^
Cyhalothrin	*C_t_* = 106.3307e^−0.0413*t*^	0.0413	16.8	0.9641
Fenpropathrin	*C_t_* = 108.9340e^−0.0363*t*^	0.0363	19.1	0.9457
Deltamethrin	*C_t_* = 108.9888e^−0.0297*t*^	0.0297	23.3	0.9470
Cyfluthrin	*C_t_* = 110.5794e^−0.0249*t*^	0.0249	27.8	0.9196
Beta-cypermethrin	*C_t_* = 107.6317e^−0.0260*t*^	0.0260	26.7	0.9501
Bifenthrin	*C_t_* = 104.1498e^−0.0112*t*^	0.0112	61.9	0.9634
