# Supplementary material for: Pathway and kinetics of cyhalothrin biodegradation by Bacillus thuringiensis strain ZS-19
Source: Sci Rep. 2015 Mar 5;5:8784. doi: 10.1038/srep08784 (PMC4350101; doi:10.1038/srep08784)
Supplement: Supplementary Information [file srep08784-s1.pdf]

Pathway and kinetics of cyhalothrin biodegradation by *Bacillus thuringiensis* strain ZS-19

Shaohua Chen[a,b*], Yinyue Deng[a,b], Changqing Chang[a,b], Jasmine Lee[b], Yingying Cheng[a], Zining Cui[a], Jianuan Zhou[a], Fei He[a], Meiying Hu[c], Lian-Hui Zhang[a,b,d*]

[a] Guangdong Province Key Laboratory of Microbial Signals and Disease Control, South China Agricultural University, Guangzhou 510642, Peoples' Republic of China

[b] Institute of Molecular and Cell Biology, Agency for Science, Technology and Research (A*STAR), 61 Biopolis Drive, Proteos, Singapore 138673, Republic of Singapore

[c] Key Laboratory of Natural Pesticide and Chemical Biology, Ministry of Education, South China Agricultural University, Guangzhou 510642, Peoples' Republic of China

[d] Department of Biological Sciences, National University of Singapore, Republic of Singapore

[*] Corresponding Author

Address: Guangdong Province Key Laboratory of Microbial Signals and Disease Control, College of Natural Resources & Environment, South China Agricultural University, Guangzhou 510642, Peoples' Republic of China

Tel: +86-20-85288229; Fax: +86-20-85280292.

E-mail: shchen@scau.edu.cn (S.Chen); lianhui@imcb.a-star.edu.sg (L.-H. Zhang).

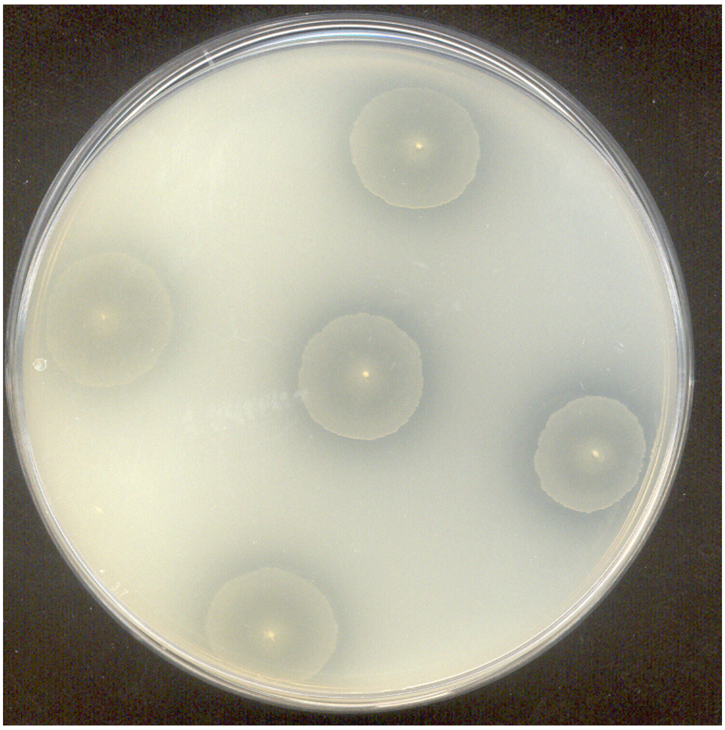

Figure S1. Clear haloes were produced around the colonies of strain ZS-19 when grown on agar plate containing 100 μg·ml^{-1} cyhalothrin

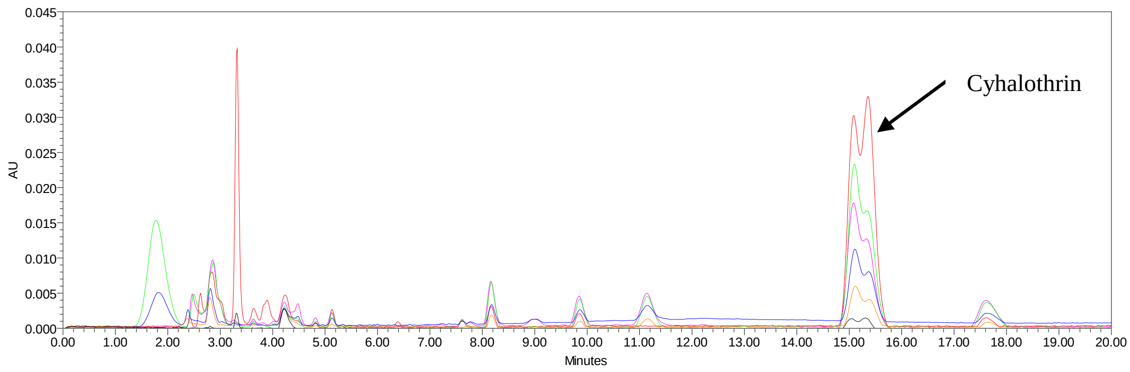

Figure S2. HPLC analysis of cyhalothrin degradation by strain ZS-19 over time

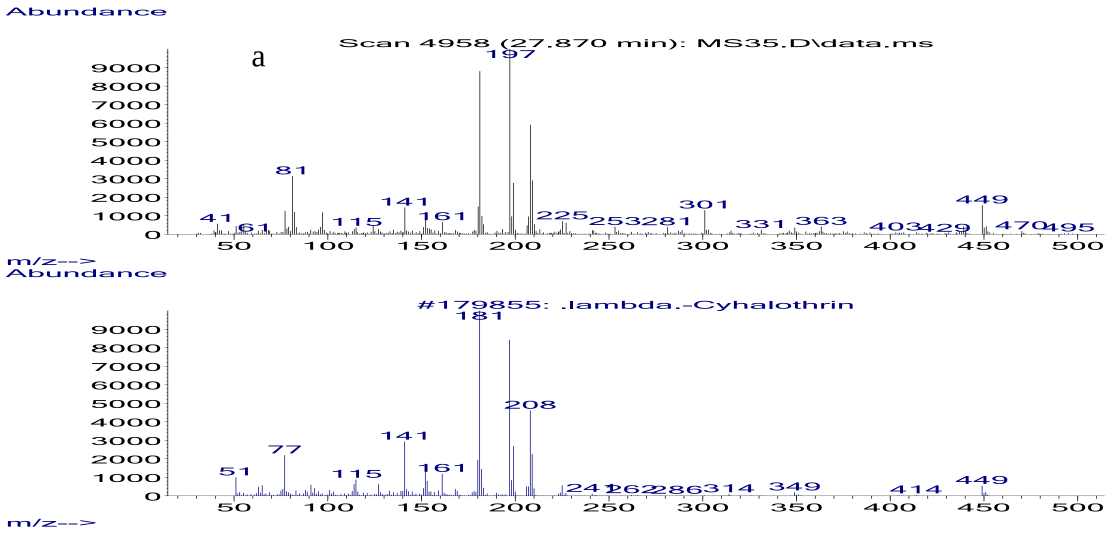
Abundance
a
Scan 4958 (27.870 min): MS35.D\data.ms
197
81
141
161
41
61
115
225
253
281
301
331
363
403
429
449
470
495
m/z-->
Abundance
#179855: .lambda.-Cyhalothrin
181
208
141
77
51
115
161
241
262
286
314
349
414
449
m/z-->

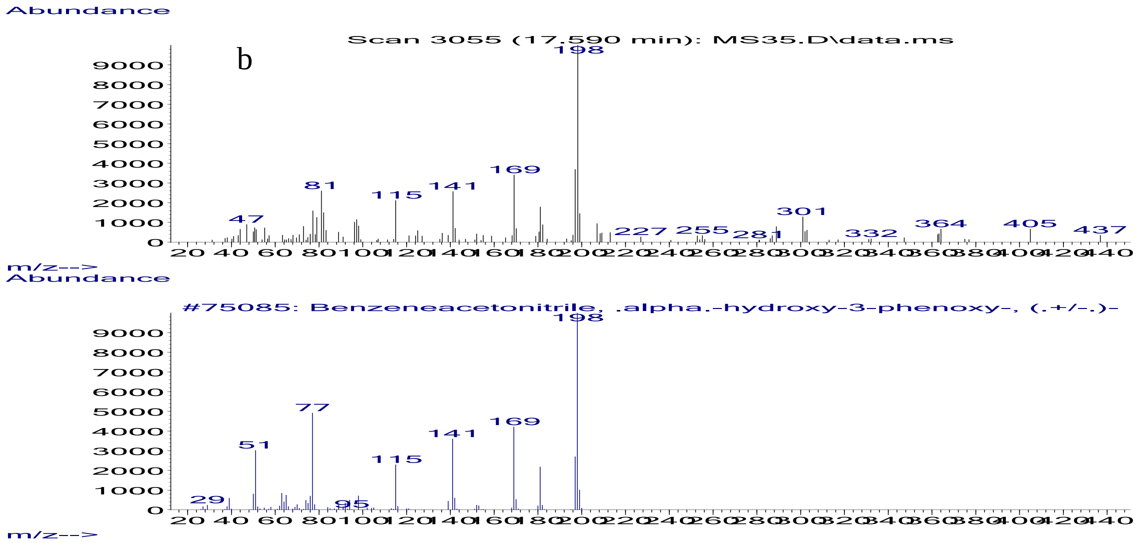
Abundance
b
Scan 3055 (17.590 min): MS35.D\data.ms
198
169
81
141
115
47
301
227
255
281
332
364
405
437
m/z-->
Abundance
#75085: Benzeneacetonitrile, .alpha.-hydroxy-3-phenoxy-, (.+/-.)-
198
77
169
141
51
115
29
95
m/z-->

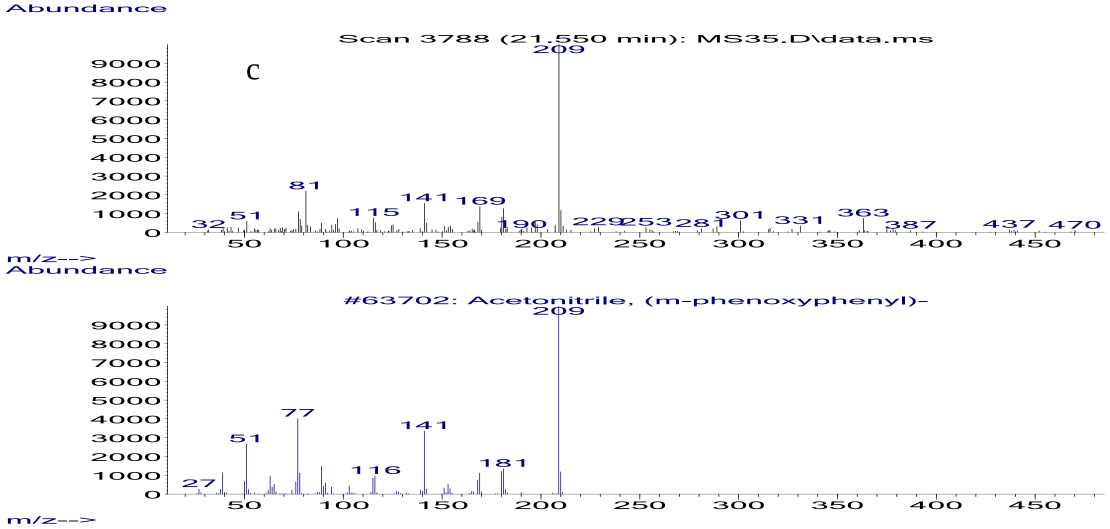
Abundance
c
Scan 3788 (21.550 min): MS35.D\data.ms
209
81
141
169
115
32
51
190
229
253
281
301
331
363
387
437
470
m/z-->
Abundance
#63702: Acetonitrile, (m-phenoxyphenyl)-
209
77
141
51
181
116
27
m/z-->

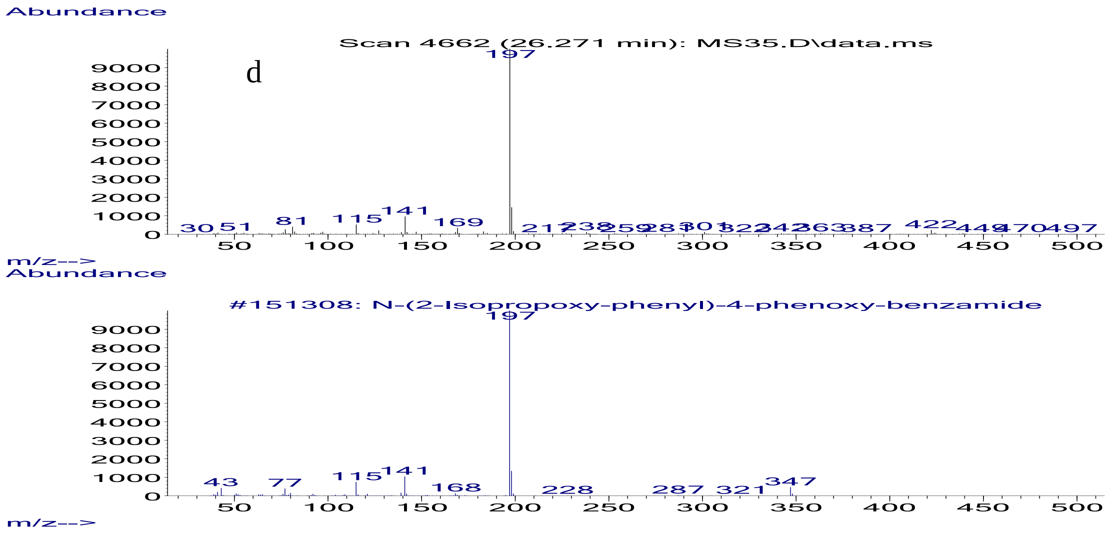
Abundance
Scan 4662 (26.271 min): MS35.D\data.ms
d
m/z-->
Abundance
#151308: N-(2-Isopropoxy-phenyl)-4-phenoxy-benzamide
m/z-->

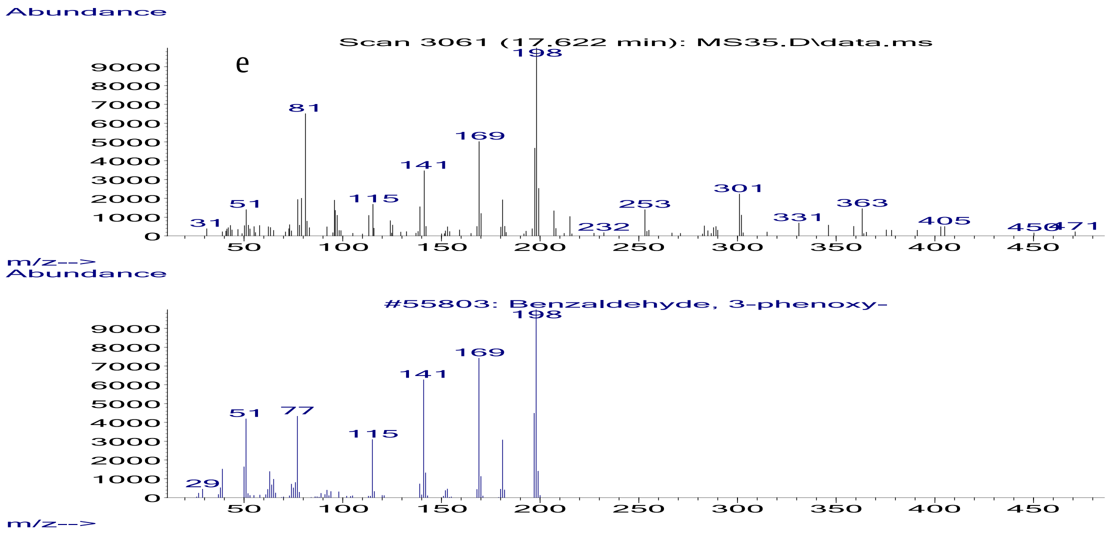
Abundance
Scan 3061 (17.622 min): MS35.D\data.ms
e
m/z-->
Abundance
#55803: Benzaldehyde, 3-phenoxy-
m/z-->

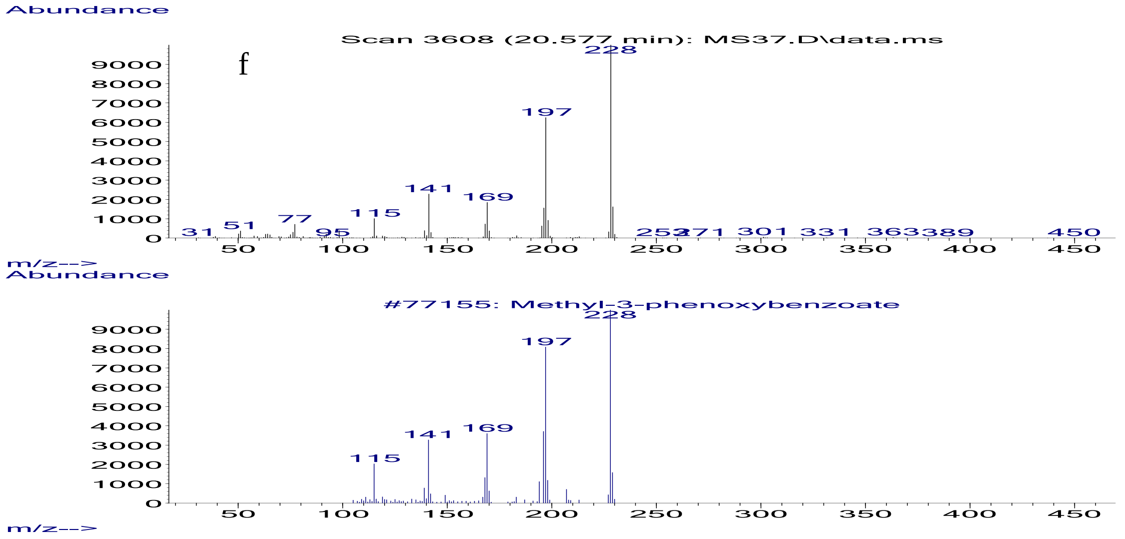
Abundance
Scan 3608 (20.577 min): MS37.D\data.ms
f
m/z-->
Abundance
#77155: Methyl-3-phenoxybenzoate
m/z-->

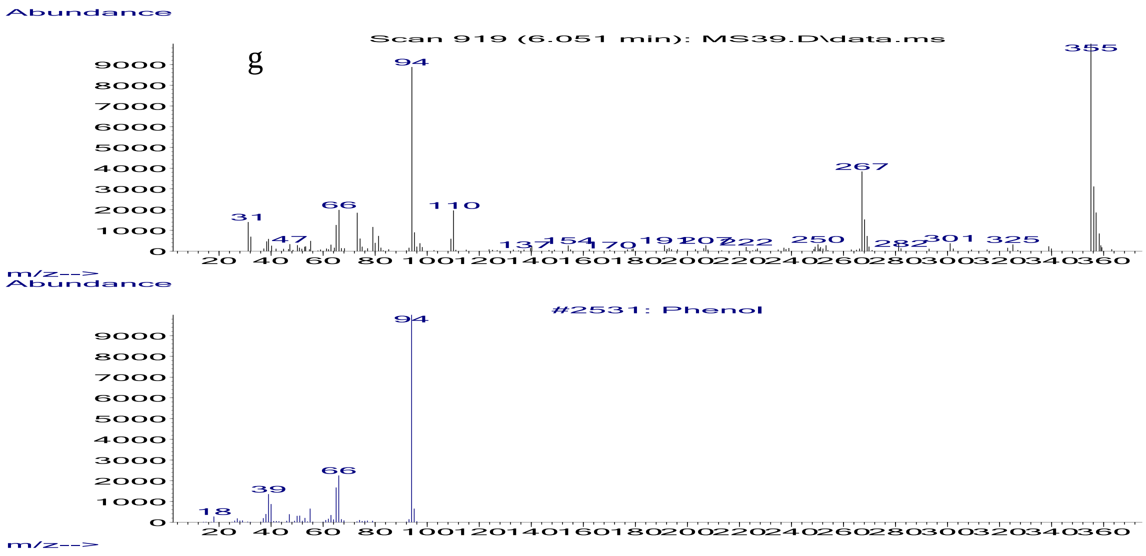

Figure S3. The mass spectra of cyhalothrin metabolites reported in the NIST library database. (a) cyhalothrin; (b) *α*-hydroxy-3-phenoxy-benzeneacetonitrile; (c) 3-phenoxyphenyl acetonitrile; (d) *N*-(2-isoproxy-phenyl)-4-phenoxy-benzamide; (e) 3-phenoxybenzaldehyde; (f) 3-phenoxybenzoate; (g) phenol.

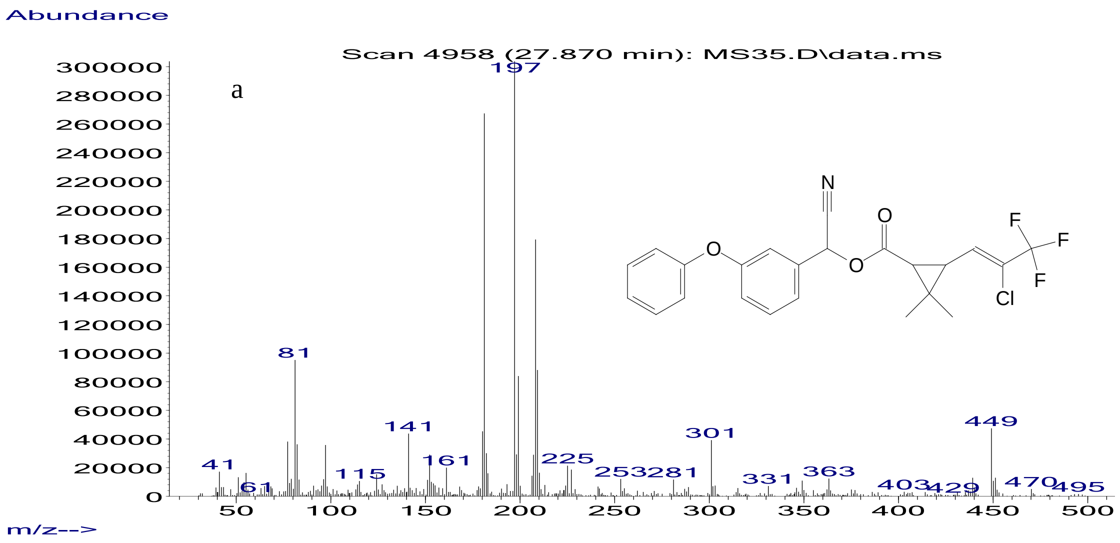
Abundance
Scan 4958 (27.870 min): MS35.D\data.ms
a
m/z-->
197
81
141
161
225
301
449
363
N
O
F
Cl

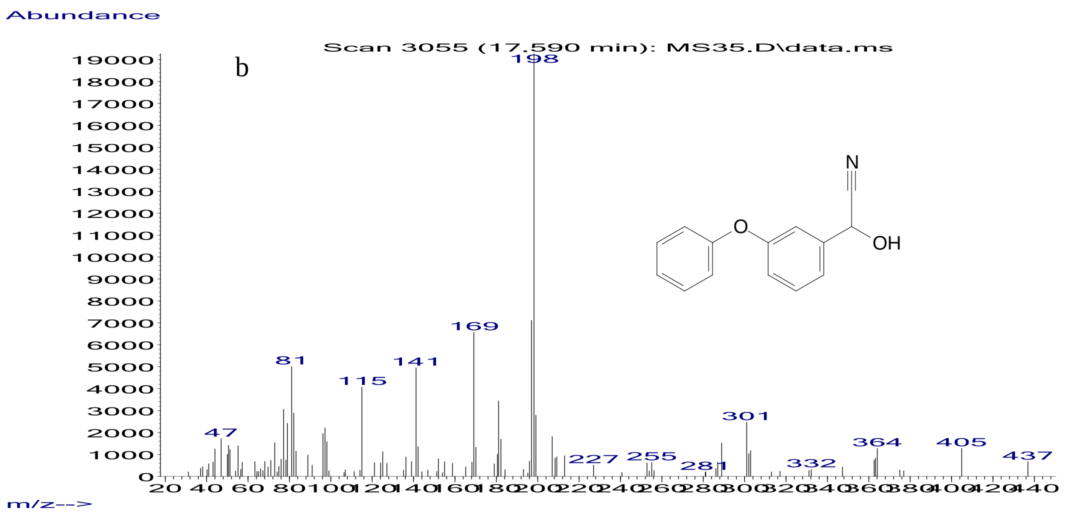
Abundance
Scan 3055 (17.590 min): MS35.D\data.ms
b
m/z-->
198
169
141
115
81
301
364
405
437
N
O
OH

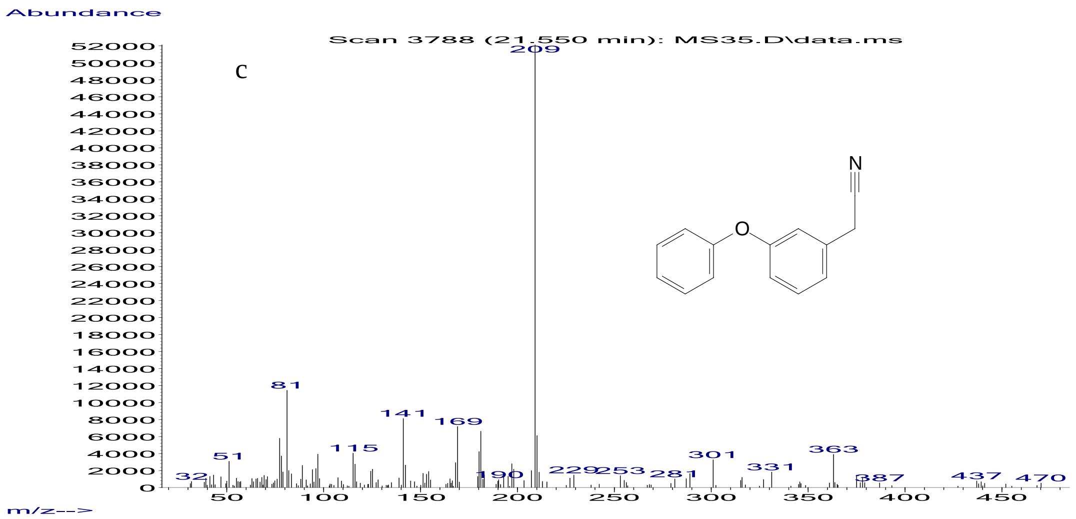
Abundance
Scan 3788 (21.550 min): MS35.D\data.ms
c
m/z-->
209
81
141
169
115
301
363
N
O

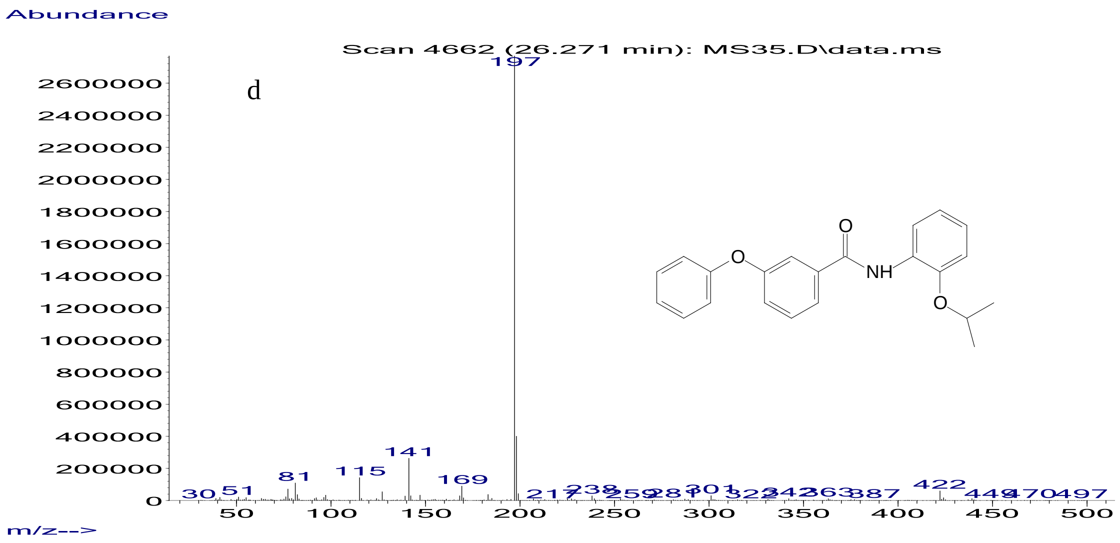
Abundance
Scan 4662 (26.271 min): MS35.D\data.ms
d
197
141
115
169
81
51
30
422
m/z-->

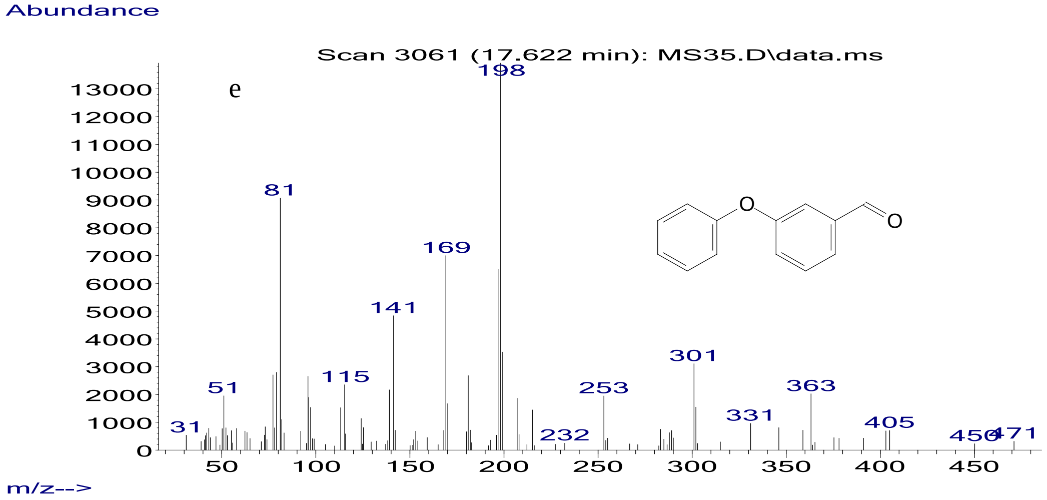
Abundance
Scan 3061 (17.622 min): MS35.D\data.ms
e
198
81
169
141
115
51
31
301
363
253
331
232
405
471
m/z-->

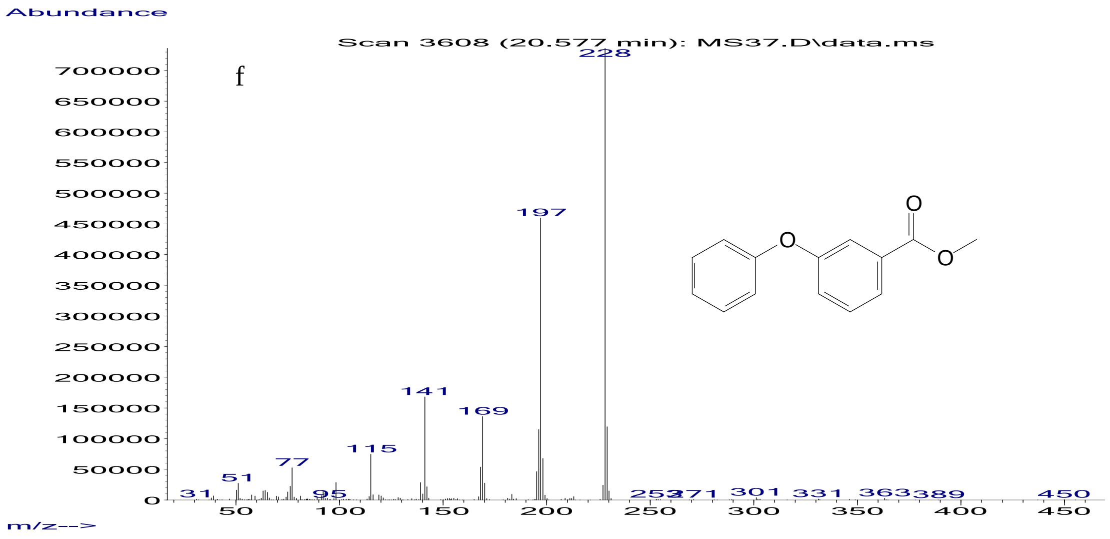
Abundance
Scan 3608 (20.577 min): MS37.D\data.ms
f
228
197
141
169
115
77
51
31
95
450
m/z-->

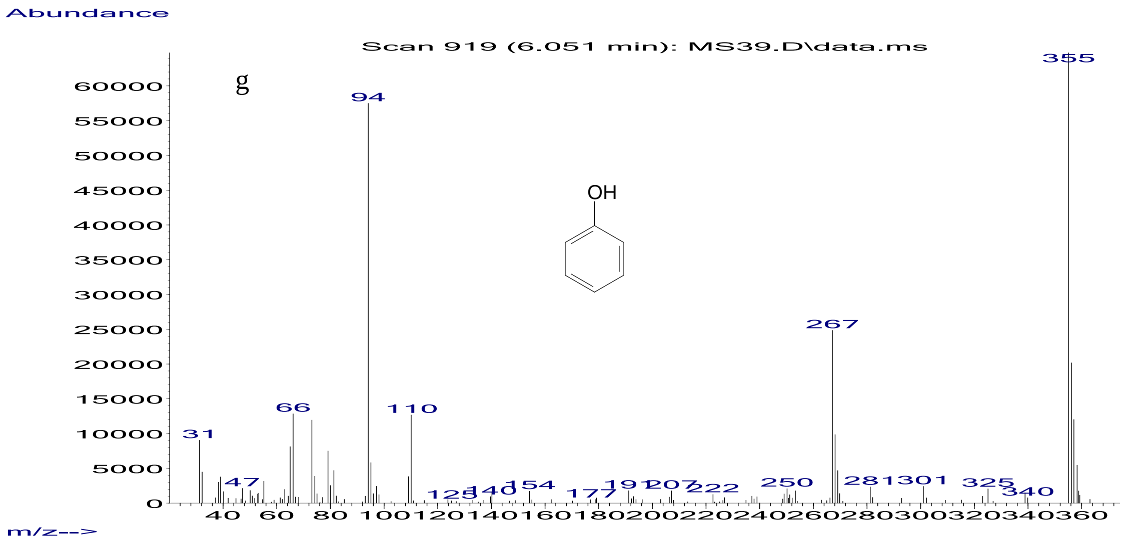

Figure S4. GC-MS analysis of the metabolites produced from cyhalothrin degradation by strain ZS-19. (a) cyhalothrin; (b) *α*-hydroxy-3-phenoxy-benzeneacetonitrile; (c) 3-phenoxyphenyl acetonitrile; (d) *N*-(2-isoproxy-phenyl)-4-phenoxy-benzamide; (e) 3-phenoxybenzaldehyde; (f) 3-phenoxybenzoate; (g) phenol.

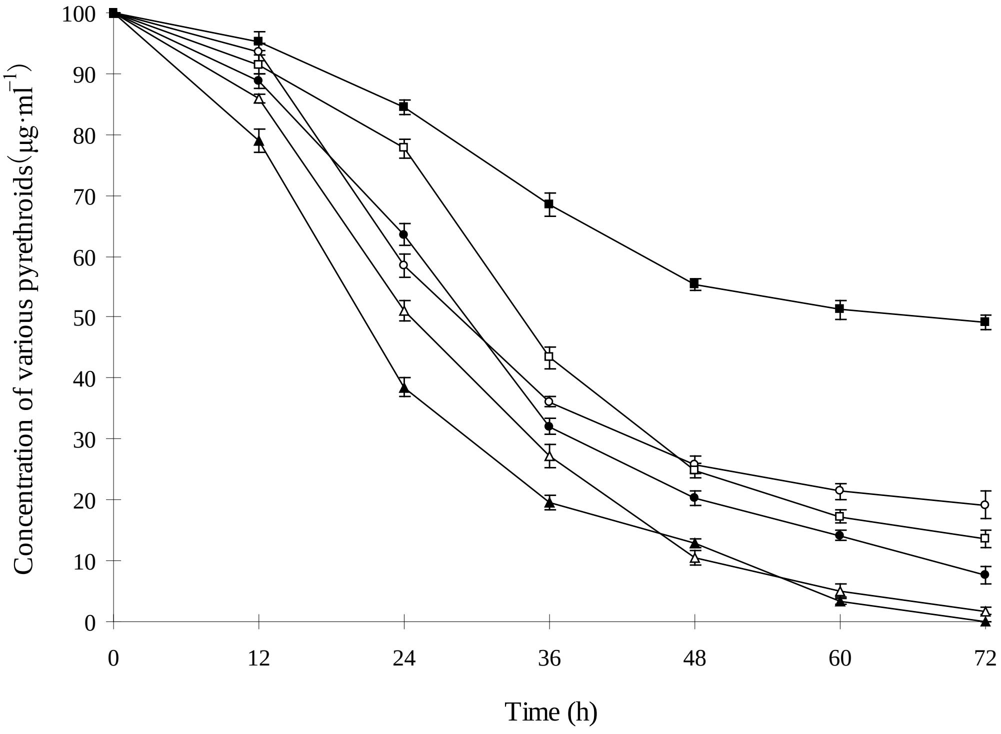

Figure S5. Degradation kinetics of various pyrethroids (100 μg·ml^{-1}) by strain ZS-19. Symbol: ▲, cyhalothrin ; △,fenpropathrin; ●, deltamethrin; ○, beta-cypermethrin; □, cyfluthrin; ■, bifenthrin
